# A Pilot Study Examining Neural Response to Pain in Adolescents With and Without Chronic Pain

**DOI:** 10.3389/fneur.2019.01403

**Published:** 2020-01-15

**Authors:** Scott A. Jones, Holly E. Cooke, Anna C. Wilson, Bonnie J. Nagel, Amy L. Holley

**Affiliations:** ^1^Department of Psychiatry, Oregon Health & Science University, Portland, OR, United States; ^2^School of Graduate Psychology, Pacific University, Hillsboro, OR, United States; ^3^Department of Pediatrics, Oregon Health & Science University, Portland, OR, United States; ^4^Department of Behavioral Neuroscience, Oregon Health & Science University, Portland, OR, United States

**Keywords:** brain, adolescents, functional MRI, posterior cingulate, default mode network

## Abstract

**Introduction:** Chronic pain is common in adolescence and is associated with both pain and prevalence of mental illness later in life. While previous functional neuroimaging work has informed knowledge of neural alterations associated with chronic pain, these findings have been primarily limited to adult samples, and it is unclear if similar patterns of altered brain activation are present in the developing adolescent brain.

**Objectives:** The purpose of this study was to pilot a noxious pressure task during functional neuroimaging to assess brain response to pain in adolescents with and without chronic pain.

**Methods:** Adolescents (ages 11–16) with (*n* = 9, 7 females) and without (*n* = 9, 7 females) chronic pain, matched on age, sex, IQ, and parental history of chronic pain, completed a noxious mechanical pressure task to assess subjective pain thresholds. This was followed by randomized presentation of subjective equivalent pressure applications (adolescents' pain 4/0–10), and two objectively equivalent pressures (0.25 and 1.5 kg/cm^2^), during functional magnetic resonance imaging, using an event-related task design.

**Results:** Findings revealed that adolescents with chronic pain demonstrated significantly greater activation in the posterior cingulate compared to controls. Further, all adolescents demonstrated significant pain-related brain response in brain regions implicated in pain neurocircuitry, as well as in several regions of the default mode network. Similar patterns of neural response were also noted during pain anticipation.

**Conclusion:** These findings are important for not only understanding the neurocircuitry involved in adolescent chronic pain, but may prove beneficial to future pain treatment efforts that seek to alter pain neurocircuitry.

## Introduction

Chronic pain is common in adolescents (1) and is associated with sleep impairments [e.g., (2)], symptoms of anxiety and depression [e.g., (3)], impairments in social development [e.g., (4)], and reduced academic success [e.g., (5)]. Prevalence and intensity of adolescent chronic pain increase with age [for review, see (1)]. Likely accompanying these changes in pain symptomology are changes in pain circuitry in the brain, as adolescence is a period of increased neuroplasticity in which functional and structural networks of the brain, including those underlying pain responsivity, undergo significant development [for review, see (6)]. Taken together, this makes adolescence a key developmental window for the study of neural response to chronic pain, as a potential means to inform intervention efforts.

Studies using functional magnetic resonance imaging (fMRI) in healthy adults have shown brain regions, including the thalamus, somatosensory cortices, and posterior insula are important for processing the location and intensity of pain, while the thalamus, anterior cingulate cortex, anterior insula, and prefrontal cortex are important for processing the emotional aspects of pain [for review, see (7)]. Further, anticipation of pain in healthy adults, even in the absence of painful stimuli, can result in brain activation in the primary somatosensory cortex, anterior insula, anterior cingulate, and medial prefrontal cortex (8). In response to mechanical stimulation, adults with chronic pain demonstrate alterations in neurocircuitry, including greater activation in primary and secondary somatosensory cortices, prefrontal cortex, inferior parietal lobe, posterior cingulate, insula, and supplementary motor cortex, when compared to healthy controls (9–12).

fMRI studies in adolescents with chronic pain are limited and have focused primarily on brain structure, or functional connectivity, not functional response to painful stimuli. Adolescents with chronic pain have thicker posterior cingulate cortices, thinner posterior parietal, and prefrontal cortices, reduced gray matter volume in the thalamus, caudate, and nucleus accumbens, and poorer white matter integrity in the dorsal cingulate, compared to healthy controls (13–15). Further, adolescents with chronic pain have less functional connectivity between prefrontal and posterior cingulate cortices and between the anterior cingulate and precuneus, but more connectivity between the caudate and precentral gyrus (13, 15). To our knowledge, only one study has investigated brain response to painful stimuli in adolescents with (*n* = 10) and without (*n* = 10) chronic pain. Findings revealed adolescents with chronic pain had lower brain activation in the thalamus, precentral gyrus, and prefrontal cortex (16). This study utilized a block design, with fixed intervals between stimuli, making it impossible to disentangle stimuli response from a pain anticipatory response, the latter of which may be indicative of an underlying pattern of neural deviation beyond what occurs during actual experiences of pain. Understanding of the patterns of neural activation in adolescents with chronic pain, both to pain stimuli and anticipation of pain, and how they differ from literature on adults, is necessary to better inform intervention efforts.

The current study adapted and piloted a noxious pressure task to elicit neural response to pain during fMRI in adolescents with and without chronic pain. This study is the first to utilize an event-related design to assess brain response in adolescents during objectively and subjectively equivalent pressure stimuli presented at variable intervals, allowing assessment of pain anticipation prior to stimulus application in adolescents. Based on previous findings, we hypothesized pressure-related activation in regions involved in the pain network (thalamus, primary and secondary somatosensory cortex, insula, cingulate, and prefrontal cortex), and that adolescents with chronic pain would demonstrate altered neural response in these regions compared to controls.

## Methods

### Recruitment and Exclusionary Criteria

Adolescents with chronic pain (*n* = 10) were recruited from an interdisciplinary pediatric pain clinic in the United States. Inclusion criteria for youth with chronic pain were: age 11–17 and a diagnosis of current chronic pain, not due to a serious chronic health condition (e.g., juvenile idiopathic arthritis, Crohn's Disease), based on physician evaluation. Youth without chronic pain (controls; *n* = 15) were recruited via community advertisements. Inclusion criteria for youth without chronic pain were: no history of chronic pain or current complaint of chronic or recurrent pain. Exclusionary criteria for participants in both groups was: left handedness (17), comorbid serious medical problems (e.g., cancer, diabetes, and head trauma), injury to hand that prevented participation in pressure task, current use of psychotropic or prescription pain medications, intellectual or learning disabilities, prenatal exposure to drugs or alcohol, and MRI contraindications (e.g., metal in body). During recruitment, adolescents and their parents provided written consent and assent, respectively, and participants received $70 for participation in the study. The study was approved by the appropriate Institutional Review Board.

### Adolescent-Report Measures

The Patient-Reported Outcomes Measurement Information System (PROMIS) Pediatric Anxiety and Depressive Symptoms Short Forms were administered to adolescents to assess psychopathological symptoms (18). Pubertal development was self-reported using the Pubertal Development Scale (PDS) Crockett Staging which ranges from stage 1 (pre-puberty) to stage 5 (post-puberty). Adolescents completed a pain questionnaire reporting on pain frequency, pain intensity, and location they experienced the “most problems with aches or pains” during the past month. The pain frequency question had 6 categories ranged from “daily” to “not at all,” and pain intensity was assessed on an 11-point numerical rating scale (NRS) from 0 (no pain) to 10 (worst pain possible). Further, total number of pain locations over the past 3 months was calculated by summing responses to a pain location checklist in which participants respond “yes” or “no” as to whether they experienced pain in each of 9 body areas.

### Parent-Report Measures

To assess family history of chronic pain, parents reported on their own history of chronic pain and current chronic pain symptoms (defined as pain present weekly or more frequently for 3 months), as well as chronic pain history in the child's other biological parent. Adolescents were categorized as having a parent with chronic pain if either of their biological parents had current or past chronic pain.

### Examiner-Administered Measures

To estimate overall intellectual functioning, adolescents were administered the 2-subtest version of the Wechsler Abbreviated Scale of Intelligence (19).

### Noxious Pressure Task

Immediately prior to the imaging session (described below), adolescents underwent a noxious pressure task to assess subjective pain response. Pressure was delivered using a custom-made fMRI-compatible device [Arbor Medical Innovations, Ann Arbor, MI] that applied pressure to the thumb of the dominate (right) hand via a 1 cm^2^ circular rubber probe attached to a hydraulically-driven piston. During this task, adolescents received variable, increasing levels of pressure stimuli (5 s stimuli, followed by 20 s of no stimuli), and were asked to rate the pain intensity on a NRS (0–10) after each stimuli (20). The task started with three cycles of 1.0, 1.5, and 2.0 kg/cm^2^ stimuli, followed by increasing pressures from 2.5 to 4.5 kg/cm^2^ (in increments of 0.25 kg/cm^2^), with 1.0 kg/cm^2^ stimuli between each increasing pressure. Participants received 12–28 stimuli; the task was terminated when subjects rated two or more consecutive stimuli as a 5/0–10 or higher. Following the completion of this task, an individualized “pain 4” was calculated, based on the average of all applied pressure with a reported pain score of 4, and the average pain intensity response on the three 1.5 kg/cm^2^ was calculated to estimate the subjective response to an “objectively equivalent” pressure stimuli.

### fMRI Assessment

**Figure 1:**
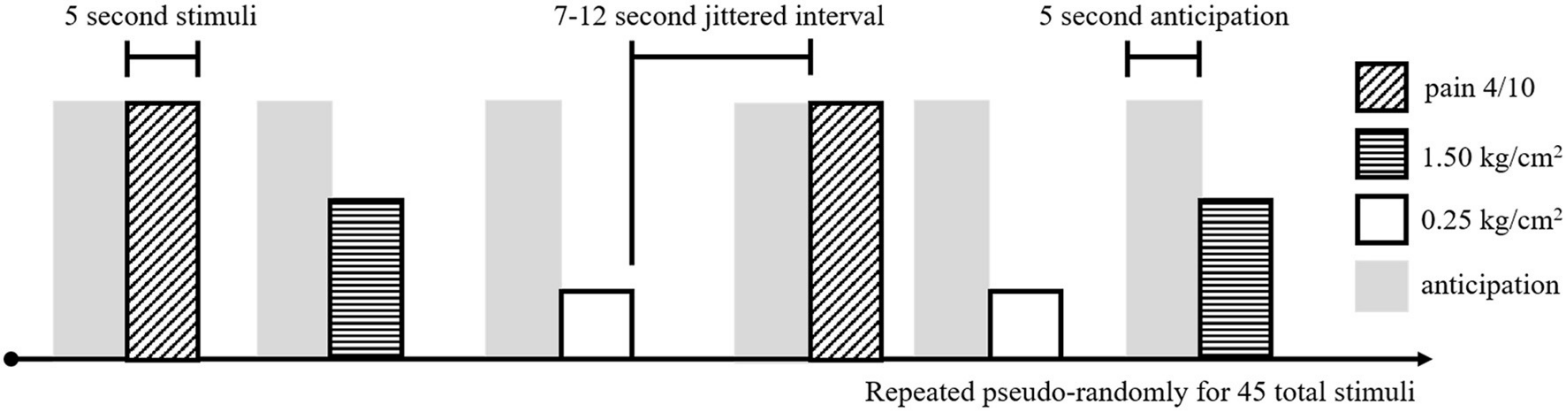
Experimental task design for the noxious pressure task during fMRI. Pain 4/10 stimuli (diagonal hashed boxes), 1.50 kg/cm^2^ stimuli (horizontal hashed boxes), and 0.25 kg/cm^2^ stimuli (open boxes) were presented in a pseudo-random order, 15 times for each stimuli, for a total of 45 pressure stimuli. Each stimuli was 5 s long and separated by a 7–12 s jittered interval of rest. Preceding each stimuli, a 5 s pain anticipation period (gray shading) was modeled.

To assess brain response to pain, adolescents received a noxious pressure paradigm (using the applied pressure device described above, with pressure applied to the right thumb) during fMRI (Figure 1). This task was adapted from a paradigm shown to evoke changes in resting state functional connectivity in individuals with fibromyalgia (21). During fMRI, participants received three types of pressure stimuli: (1) a light touch (non-painful) pressure of 0.25 kg/cm^2^, (2) an objectively equivalent pressure of 1.5 kg/cm^2^, and (3) a subjectively equivalent pressure (pain 4), individually-adjusted to match the pressure at which participants reported a 4/0–10 on the NRS in the previous task. Fifteen of each stimuli were randomly presented for 5 s each, with a jittered intertrial interval of 7–12 s, for a total of 45 stimuli (10:40 min). During administration of the pressure task (and the preceding resting state scan—see below), participants were presented with a white fixation cross on a black background and were instructed to “focus on the plus sign, think about whatever they want, and remain as still as possible.”

### Image Acquisition

Participants were scanned on a 3.0 Tesla Siemens Magnetom Prisma with a 20-channel head/neck coil. For image registration, high-resolution T1-weighted MPRAGE structural scans were collected in the sagittal plane [time to repetition (TR) = 2,300 ms, time to echo (TE) = 3.61 ms, inversion time (TI) = 900 ms, flip angle = 10°, voxel size = 1 × 1 × 1.1 mm, acquisition time 9:14 min]. During administration of the noxious pressure task, functional T2^*^-weighted gradient echo-planer images were collected axially and parallel to the anterior commissure-posterior commissure plane (TR = 2,000 ms, TE = 30 ms, flip angle = 90°, voxel size = 3.75 × 3.75 × 3.8 mm, acquisition time = 10:42 min). Additional functional T2^*^-weighted gradient echo-planer images were also collected during rest (TR = 2,500 ms, TE = 30 ms, flip angle = 90°, voxel size = 3.75 × 3.75 × 3.8 mm, acquisition time = 2 runs of 5:17 min each).

### Image Pre-processing

Images were preprocessed using Analysis of Functional NeuroImages (AFNI; v 18.3.01) (22), via methods described previously (23). This included, visual inspection for scanner-related artifacts, removal of the first 3 volumes, within-run volume registration, co-registration of functional images to structural images, and transformation to Talairach space using linear and non-linear registration. Functional images were smoothed using a 6.0 mm full-width half-maximum Gaussian kernel, normalized to whole-brain signal to obtain percent signal change, and resampled to 3 × 3 × 3 mm. Finally, to compare brain response to pain to brain response during rest (see details below) functional images collected during the noxious pain task and rest scan were temporally concatenated prior to generating the first-level model.

### Individual-Level Model

To assess brain response to pressure stimuli, the first-level model included regressors representing the three stimulus types (0.25, 1.5 kg/cm^2^ and pain 4), with stimulus times corresponding to the onset time of each stimuli, and the duration encoded as the entire time pressure was applied (~5 s), convolved with a gamma-variate hemodynamic response function. Additionally, to model pain anticipation, an anticipation regressor was generated with a duration of 5 s and a stimulus onset time occurring 5 s prior to each stimulus onset. Given that AFNI's intrinsic baseline includes all remaining un-modeled time (which may be confounded by residual brain response to pain and/or un-modeled pain anticipation, and which may differ based on chronic pain history), a series of pseudo regressors were created using the rest sequence immediately preceding the pressure task. To minimize the risk of incidentally capturing differential non-task-related fluctuations in brain response between the two sequences (pressure task and rest), these rest regressors were identical to the onset and duration of the pressure stimuli modeled during the noxious pressure task. Additional nuisance regressors were included to model linear drift and motion using the 6 parameters obtained during volume registration. Finally, binary motion censoring files were created with volumes censored if they had a framewise displacement > 0.7 mm or if there were five or fewer contiguous frames of good data (24). All pressure, rest, anticipation, and nuisance regressors were included in a single general linear model (GLM) for each subject, and were adjusted according to the volume censoring file provided. The design matrix for the individual-level GLM was generated using AFNI's 3dDeconvolve and subsequently fit to the functional data using AFNI's 3dREMLfit. Using the task regressors, four contrasts of interest were created: 0.25 kg/cm^2^ vs. rest, 1.5 kg/cm^2^ vs. rest, pain 4 vs. rest, and anticipation vs. rest.

### Additional Exclusionary Criterion

Prior to group-level analyses, an additional 7 subjects were excluded. Two subjects were excluded from analysis, as they completed the entire pressure task without rating any pressures as a 4/0–10, and thus an appropriate pain 4 could not be calculated. Additionally, five subjects were excluded from analysis as they had either ≥ 5/15 of any pressure stimuli censored, or <5 min of acceptable resting state data after censoring (based on previous unpublished findings regarding the number of repetitions necessary to reliability model the hemodynamic response for a particular stimuli). This resulted in a final sample of 9 adolescents with chronic pain and 9 controls.

### Group-Level Analyses

Participant characteristics were assessed for normality and compared between groups using R (version 3.4.2). Independent samples *t*-tests were used to test for group differences in age, IQ, pain rating during the 1.5 kg/cm^2^ condition, and pressure applied during pain 4 condition. Chi square tests were used to assess group differences in sex, ethnicity, and parental history of chronic pain. Anxiety and depression scores, pubertal development, and reported frequency and intensity of pain were compared between chronic pain adolescents and controls using Mann-Whitney-Wilcoxon tests.

To assess the effects of noxious pressure on brain response, a 2 (group: chronic pain and control) by 3 (pressure condition: 0.25, 1.5 kg/cm^2^, pain 4) mixed factorial analysis of variance (ANOVA) was conducted using AFNI's 3dMVM (25). To exclude the potential of presumably false findings in the white matter and/or ventricles, and to reduce the number of voxel-wise comparisons, this was fit voxel-wise in a standardized gray matter mask (generated via tissue segmentation of the standard Talairach template used during spatial registration), with a voxel-wise threshold of *p* < 0.001 and a cluster-forming threshold of α < 0.05 (11 contiguous voxels). This cluster forming threshold was calculated using AFNI's 3dClustsim using the auto-correlation function (ACF) parameters obtained from the individual-level GLM residuals. This voxel-wise threshold, and method for calculating the cluster-forming threshold, has been recently shown to result in false positive rates at or below the widely accepted 5% cutoff for event-related designs (26).

In all regions where a significant effect of condition, group, or condition-by-group interaction was present, mean brain response was extracted, and *post-hoc* paired and independent-samples *t*-tests (Bonferroni-corrected) were carried out in R. Further, in regions where a significant group effect or group-by-condition effect was present, mean brain response was extracted, and *post-hoc* one-sample *t*-tests (Bonferroni-corrected) were used to assess significant differences from rest for each group. These one-sample comparisons were carried out largely for interpretive purposes, as a positive group differences may be driven by greater activation in one group compared to rest, or by less deactivation in that same group compared to rest. Finally, for all regions where there were significant group, condition, or group-by-condition effects, brain response during anticipation was extracted and compared between group and condition.

## Results

### Differences Between Adolescents With and Without Chronic Pain

Participant demographics and pain characteristics are presented in Table 1. Adolescents with chronic pain and controls did not differ based on age, sex, ethnicity, IQ, or presence of a biological parent with chronic pain. Adolescents with chronic pain reported an average of 5 pain locations (SD = 1.73) with most painful parts of the body being abdomen (*n* = 3), face/head (*n* = 2), back (*n* = 2), and leg/foot (*n* = 2). Average pain ratings during the 1.5 kg/cm^2^ stimuli across adolescents ranged from 0.33 to 4.33/10, and the pressure necessary to reach a pain 4/10 ranged from 1.5 to 4.5 kg/cm^2^; however, there were no group differences in these measures.

**Table 1:** Sample characteristics.

	**Chronic pain (*n* = 9) Mean (SD)**	**Control (*n* = 9) Mean (SD)**
Sex (male/female)	2/7	2/7
Ethnicity		
White	7	5
American Indian/Alaskan Native	1	0
Hispanic/Latino (of any race)	1	2
Native Hawaiian/Pacific Islander	0	2
Age	15.62 (1.17)	15.22 (1.33)
Pubertal development		
Pre-pubertal	0	0
Early-pubertal	0	0
Mid-pubertal	4	4
Late-pubertal	2	1
Post-pubertal	2	4
Unknown	1	0
IQ	109.89 (10.20)	104.44 (18.43)
Parental chronic pain (yes/no)	7/2	5/4
Anxiety scores^*^	1.00 (0.79)	0.43 (1.01)
Depression scores^*^	0.90 (0.89)	0.28 (0.70)
1.5 kg/cm^2^ pain rating	2.62 (1.03)	1.92 (0.98)
Pain 4 pressure	2.64 (0.96)	2.83 (0.85)
Pain intensity^**^	4.67 (2.00)	1.67 (1.73)
Pain frequency^***^		
Daily	6	0
4–6 days/week	2	0
2–3 days/week	0	1
Weekly	0	0
1–3 times/month	1	6
Not at all	0	2

* *p < 0.05*,

** *p < 0.01*,

*** *p < 0.001 between adolescents with chronic pain and controls*.

As expected, adolescents with chronic pain reported significantly more frequent (*U* = 4, *p* < 0.001) and intense (*U* = 8, *p* < 0.01) pain. Further, adolescents with chronic pain also reported greater levels of anxiety (*U* = 16.5, *p* < 0.05) and depression (*U* = 14.5, *p* < 0.05) than controls; thus, anxiety and depression scores were tested as covariates *post-hoc*.

**Figure 2:**
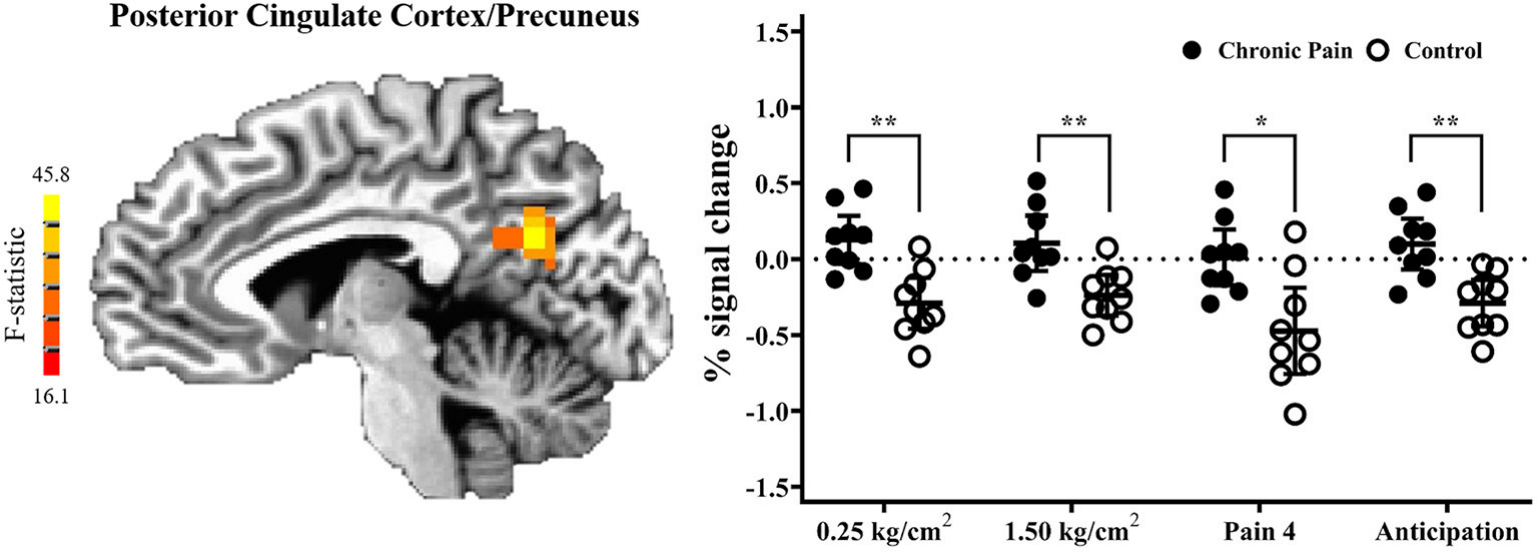
Adolescents with chronic pain, compared to controls, demonstrated significantly greater activation across all task conditions, in the posterior cingulate cortex/precuneus (voxel-wise *p* < 0.001 corrected). **(Left)** F-statistics for the main-effect of group, from the voxel-wise analysis, are depicted in the sagittal plane (*x* = −5). **(Right)** Average percent signal change, compared to rest, is plotted for each pain stimuli condition, with individual means plotted for adolescents with chronic pain (solid circles) and controls (open circles), with line and whisker plots representing group means and 95% confidence intervals for each condition. For simple effects: ^*^adjusted *p* < 0.05, ^**^adjusted *p* < 0.01.

Results of the voxel-wise analysis revealed no regions where there was a significant group-by-pressure condition interaction. However, there was a significant main-effect of group, such that adolescents with chronic pain had greater activation than controls across all pressure stimuli, in the posterior cingulate cortex/precuneus (*p* < 0.001 corrected) (Table 2 and Figure 2). *Post-hoc* tests revealed that there were significant group effects in all conditions [0.25 kg/cm^2^: *t*(16) = 4.187, adjusted *p* < 0.01; 1.5 kg/cm^2^: *t*(16) = 3.480, adjusted *p* < 0.01; pain 4: *t*(16) = 3.302, adjusted *p* < 0.05]. Further, when collapsing across all three pain conditions, this effect was driven by significant deactivation in control adolescents [*t*(8) = 6.182, adjusted *p* < 0.001], when compared to rest. Further, adolescents with chronic pain also demonstrated greater activation during pain anticipation compared to controls in this region [*t*(16) = 3.964, *p* < 0.01]. Finally, this group effect remained significant when controlling *post-hoc* for anxiety and depression scores.

**Table 2:** Significant regions of interest from voxel-wise analysis.

	**Peak effect (TLRC)**			**Cluster size**	**F-statistic**		**Eta-squared^a^**	
**Primary region(s)**	**X**	**Y**	**Z**	**(voxels)**	**Peak**	**Average**	**Peak**	**Average**
**MAIN-EFFECT OF GROUP**								
Bilateral posterior cingulate cortex/precuneus	−4	−61	22	65	45.8	24.9	0.45	0.38
**MAIN-EFFECT OF PRESSURE CONDITION**								
R primary somatosensory/motor cortex	8	−37	61	163	35.3	13.1	0.33	0.18
L primary somatosensory/motor cortex	−16	−28	61	76	17.3	11.5	0.32	0.19
L posterior parietal lobe	−25	−52	43	32	15.6	10.3	0.25	0.14
L culmen	−25	−28	−20	24	13.4	10.7	0.17	0.13
L precuneus	−25	−67	34	13	15.8	11.2	0.19	0.16
R precuneus	17	−73	49	13	14.4	11.1	0.25	0.19

L, left; R, right;

a *Eta-Squared values are Generalized Eta-Squared values, which were calculated voxel-wise when running analyses with 3dMVM (25)*.

### Differences Between Pressure Task Conditions

**Figure 3:**
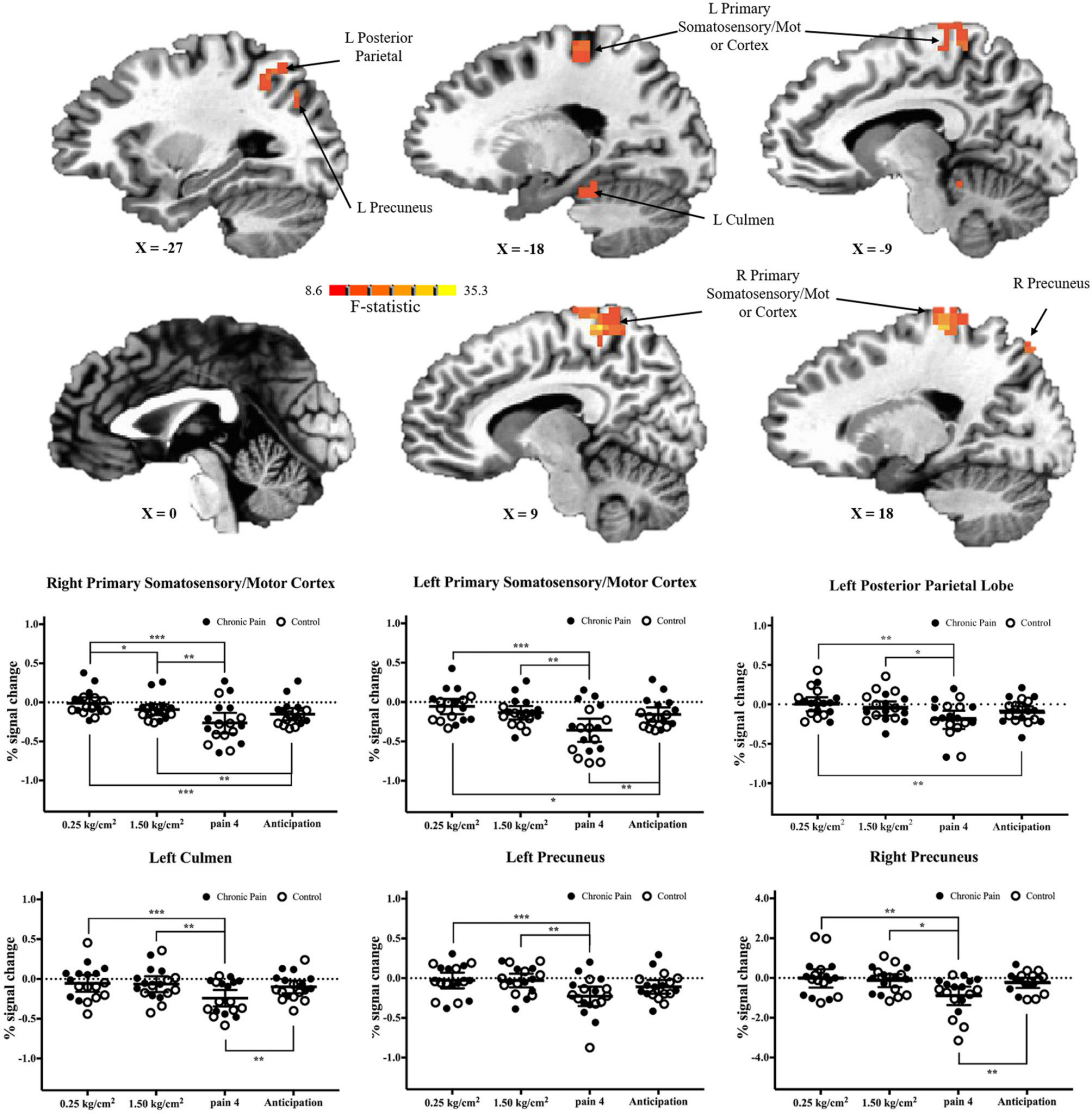
Significant main-effects of task condition, collapsed across adolescents with chronic pain and controls. **(Top)** F-statistics for the main-effect of task condition, from the voxel-wise analysis, are depicted in the sagittal plane. **(Bottom)** Average percent signal changed, compared to rest, is plotted for each condition, collapsed across adolescents with chronic pain (solid circles) and controls (open circles). Line and whisker plots represent group means and 95% confidence intervals. For simple effects: ^*^adjusted *p* < 0.05, ^**^adjusted *p* < 0.01, ^***^adjusted *p* < 0.001.

There were six clusters located in the bilateral primary somatosensory and motor cortices, the bilateral precuneus, the left posterior parietal lobe, and the left culmen, where there was a significant main effect of pressure condition (*p* < 0.001 corrected) (Table 2 and Figure 3). In all clusters, there was significantly more deactivation (compared to rest) in response to pain 4 stimuli, than in response to both the 0.25 kg/cm^2^ [all *t*(17) ≥ 3.778; adjusted *p* < 0.01] and 1.5 kg/cm^2^ stimuli [all *t*(17) ≥ 2.829; adjusted *p* < 0.05].

When comparing brain activation during anticipation to each pain condition, in all regions, average brain (de)activation to anticipation was between the 0.25 kg/cm^2^ and pain 4 conditions. That is, in regions including the primary somatosensory and motor cortices and posterior parietal lobe, there was significantly more deactivation during anticipation compared to the 0.25 kg/cm^2^ stimuli [all *t*(17) ≥ 2.763, adjusted *p* < 0.05]; meanwhile, in the primary somatosensory and motor cortices, left culmen, and right precuneus there was significantly less deactivation during anticipation compared to the pain 4 stimuli [all *t*(17) ≥ 3.446, adjusted *p* < 0.01].

## Discussion

This preliminary study investigated neural response to pain and pain anticipation in a group of adolescents with and without chronic pain using a pressure task with an event-related design. As hypothesized, we found that adolescents with chronic pain had significantly greater brain response in the posterior cingulate cortex/precuneus, compared to controls, as has been shown previously in adults (11). Further, across both groups, we found significant pain-related differences in brain response in the primary somatosensory cortex (postcentral gyrus), primary motor cortex (precentral gyrus), posterior parietal lobe, precuneus, and culmen suggesting that the noxious pressure task can successfully induce differential pain vs. rest brain response in this age group. Lastly, this is the first study to demonstrate a pattern of neural response during pain anticipation in an adolescent sample.

In line with previous studies in adults (11), in the posterior cingulate cortex, control adolescents demonstrated significant deactivation, while chronic pain adolescents showed no significant difference between pain stimuli and rest. In addition to commonly being implicated in pain processing (27), the posterior cingulate and precuneus serve as a key nodes in the default mode network, a brain network thought to be deactivated during external tasks and activated when engaged in internally driven processes, such as self-reflection, memory retrieval and envisioning the future (28, 29). Deactivation during pain processing (compared to rest) in control adolescents may suggest less internally driven thought (i.e., self-referential thought or rumination) and increased external attention to the pressure stimuli, as compared to adolescents with chronic pain. Notably, this pattern of differential activation persisted not only across all levels of pressure stimuli, but also during the anticipation phase preceding pain stimuli. This suggests that, compared to controls, adolescents with chronic pain may have stimulus-independent, sustained impairments in default mode network disengagement in the presence or anticipation of potentially painful stimuli, similar to disruptions in the default mode network during rest seen in adults with chronic pain (30). It is possible this persistent alteration in adolescent neurocircuitry could lead to a re-wiring of the brain during this vulnerable developmental period, and explain both the continuation of pain into adulthood, as well as future negative mental health outcomes, which implicate this circuitry.

In addition to differences between adolescents with chronic pain and controls, we found significant pain-related deactivation across both groups in the primary somatosensory and motor cortices, the precuneus, the posterior parietal lobe, and the culmen. As noted above, some of these regions (i.e., precuneus and posterior parietal cortex) are thought to be part of the default mode network (31). As such, significant deactivation during pain 4 stimuli may represent greater suppression of this network, as cognitive resources are redirected toward the external stimuli. However, deactivation during pain 4 stimuli (compared to 0.25 and 1.50 kg/cm^2^ stimuli) in the primary somatosensory and motor cortices is unusual and runs contrary to our hypotheses based on previous literature in adults. While the majority of existing studies suggest that activation in this region is positively associated with degree of pressure/pain applied, it must be noted that this is not the first study to demonstrate the opposite effect. For example, a previous study in healthy adults found significant deactivation in primary somatosensory, motor and supplementary motor areas during application of a painful stimuli, albeit irrespective of degree of pressure applied (32). One possible explanation for the pattern of deactivation found in our study is neural habituation as a result of receiving several pain stimuli outside of the scanner, prior to assessment via fMRI. For example, habituation of brain response to painful laser stimuli in the primary somatosensory cortex (as well as the insula and anterior cingulate) has been previously demonstrated across a single imaging session in healthy adults (33). Similarly, a decrease in brain activation in the postcentral gyrus across three phases of noxious electric stimulation has also been observed (34). Finally, in a study testing neural response to electric stimulation before and after a conditioning session (to induce habituation), the authors found a significant decrease in activation between the two session in several pain-related regions, and more importantly, found significant overall levels of deactivation in the primary somatosensory cortex and parts of the parietal cortex post-habituation (35). As such, it is possible that deactivation in pain-related regions in our study, as well as a lack of significant results in other key pain network nodes (e.g., the insula) may be due to in part to neural habituation resulting from the out-of-the-scanner task that preceded fMRI. Follow-up studies looking at the neural response to mechanical pressure across a longer period of time will be necessary to provide more evidence for this notion.

With regard to pain anticipation, we found that the left culmen and right precuneus showed levels of activation significantly different than during pain 4 stimuli, and comparable to 1.50 and 0.25 kg/cm^2^ stimuli, suggesting that pain-related activity differences between pressure stimuli in these regions may be specifically in response to the pressure application. Conversely, in regions such as the right primary somatosensory and motor cortex and posterior parietal lobe, brain activation during anticipation was significantly different than 1.50 and/or 0.25 kg/cm^2^ stimuli, but comparable to pain 4 stimuli. This is similar to findings in adults (8) and suggests that activation of pain neurocircuitry doesn't necessitate an actual stimulus, and anticipation alone may be sufficient to elicit a neural response. While these findings suggest our anticipation regressors were able to help distinguish brain regions demonstrating significant anticipation-related signal from those that do not, results in other regions are less clear. In the left primary somatosensory/motor cortex, brain deactivation during anticipation was significantly greater than in response to 0.25 kg/cm^2^ stimuli, but significantly less than compared to pain 4 stimuli. Meanwhile, in the left precuneus, brain response during anticipation was not statistically different from brain response to any other stimuli. In these regions it is unclear if our regressors captured brain response to pain anticipation (and this response is simply reduced compared to brain response during stimuli presentation), or if perhaps this anticipation regressor is actually reflecting carryover brain response from the stimuli proceeding this regressor (36). Future studies, with improved task designs (see below) will be necessary to further probe the role of pain anticipation.

While the findings of this study provide information regarding neural response to pain in adolescence, they are preliminary, and several limitations should be considered. First, because this was a pilot study, due to the small sample size we were unable to examine differential neural responses to pain by pain location. Similarly, we were unable to examine sex differences in neural responses to the pain task. This is an important avenue of future research, as female adolescents have a higher prevalence of chronic pain, experience more subjective pain-related disability than male adolescents (37), and may potentially have different underlying neurobiological profiles. Lastly, the demographics of this sample (largely white non-Hispanic with an IQ in the average range) represent a sampling bias that will need to be addressed by future studies to assess the generalizability of these findings to other demographics.

In addition to sample-related limitations, there were several notable limitations to our task design. First, significant data loss/exclusion occurred due to motion during the scan, and participants reached the upper-limit of pressure applied during assessment of pain 4. While head motion during scanning, particularly in an adolescent population, is to a degree unavoidable, raising the upper limit of the out-of-the-scanner noxious pressure task (to ensure pain 4 is captured for all adolescents) is a change easily implemented in future studies. Second, in the current task, no cues (visual or auditory) were provided prior to or during pressure stimuli. As such, during early analyses of this task, we found significant patterns of activation occurring in the pain network during rest periods between pressure stimuli, likely due to AFNI's intrinsic baseline including un-modeled pain anticipation and carry over from previous pain stimuli, which notably may differ by group status. To account for this, we attempted to model anticipation response using the 5 s preceding stimulus onset and used regressors modeled during the preceding resting state scans as a baseline from which to contrast signal related to pain stimuli. While the anticipation results in our pressure-related findings suggest we were able to capture anticipation signal in several regions, in other regions the results are less clear. In future studies using similar tasks, the inclusion of a visual/auditory cue preceding pain stimuli and longer inter-stimulus intervals, to allow full recovery from the previous stimuli, will allow for better assessment and more accurate modeling of pain-related and pain anticipation brain response.

In conclusion, results of this study provide support for using a novel pressure task with an event-related design to examine neural responses to pain in adolescents with and without chronic pain, though many task-related improvements are necessary. Findings revealed adolescents demonstrated significant pain-related deactivation in key pain-related regions of the brain, as well as in several regions of the default mode network. Compared to controls, adolescents with chronic pain demonstrated significantly greater activation in the posterior cingulate. Further, similar pain-related brain response was also noted during pain anticipation in many of these regions and may represent a pattern of chronically altered pain neurocircuitry. While many of these findings are line with previous literature, particularly in adults, future studies are necessary to confirm and expand upon these findings and further investigate the role of pain anticipation. This work is not only important for understanding the neurocircuitry involved in adolescent chronic pain, but may prove beneficial to future pain treatment efforts. As previous studies have reported changes in functional brain response following physical-biobehavioral pain treatment in adolescents (38), better understanding of the networks altered in adolescents with chronic pain may help improve treatment strategies and help reduce the rates of future pain disorders and associated psychopathology.

## Data Availability Statement

The datasets generated for this study are available on request to the corresponding author.

## Ethics Statement

The studies involving human participants were reviewed and approved by OHSU IRB. Written informed consent to participate in this study was provided by the participants' legal guardian/next of kin.

## Author Contributions

SJ, AW, BN, and AH contributed to the conception and design of the study, and development of the pressure task. SJ completed all MRI data processing and analyses. SJ, HC, and AH contributed to drafting the manuscript. All authors contributed to the interpretation of the findings, revision of the manuscript, and have read and approved the submitted version.

### Conflict of Interest

The authors declare that the research was conducted in the absence of any commercial or financial relationships that could be construed as a potential conflict of interest.
